# Designing social media content recommendation algorithms for societal good

**DOI:** 10.1111/nyas.15359

**Published:** 2025-05-05

**Authors:** Jana Lasser, Nikolaus Poechhacker

**Affiliations:** ^1^ IDea_Lab University of Graz Graz Austria; ^2^ Complexity Science Hub Vienna Vienna Austria; ^3^ University of Vienna Vienna Austria

**Keywords:** civic discourse, content recommendation, Digital Services Act, social media

## Abstract

In the face of mounting evidence for a relationship between social media platforms and detrimental societal outcomes such as polarization, the erosion of trust in institutions, and the spread of misinformation, this perspective argues for the design of alternative content recommendation algorithms that serve the societal good and a lively democratic discourse. We propose to approach the design of content recommendation algorithms through the lens of fostering a healthy civic discourse, which serves to identify dimensions of relevance to guide the development of content recommendation algorithms. This approach lends alternative content recommendation algorithms legitimacy by being rooted in the EU's novel Digital Services Act and by aligning content recommendation with democratic values. We explore the trade‐off between interventions in content recommendation and freedom of expression and propose a research agenda that uses approaches from multistakeholder metric construction and scenario‐based risk assessment to find situation‐dependent just balances between individual rights and societal outcomes.

## Graphical abstract

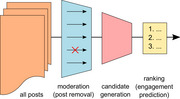

## SOCIAL MEDIA PLATFORMS AS DIGITAL TOWNSQUARES

Social media platforms increasingly serve as “digital townsquares,” virtual places where people share information and discuss; in 2020, 57% of EU citizens aged 16–74 used social media platforms, while for the current generation of young adults aged 16–24, this number is as high as 87%.^1^ However, numerous studies have now identified a role of social media in the deterioration of social cohesion, such as declining trust in institutions, increasing populism, and growing polarization (see Ref. 2 for a recent review). Facebook, for instance, facilitates the spreading of extreme viewpoints, which has been linked to an increase in violent hate crimes.^3^, ^4^ Social media use can decrease social trust,^5^ while broadband internet access in general has no such effect.^6^ It can also erode political knowledge if users expect to be presented with important information by default.^7^ While social media exposure in ethnically polarized settings can improve outgroup attitudes,^8^ the detrimental effects on civic discourse are clear for established democracies.^2^ Against this backdrop of evidence, it becomes increasingly clear that societies should play a more active role in shaping social media platforms to address their detrimental effects.

In the last few years, emerging research dedicated to the study of alternative content recommendation algorithms has proposed a number of designs for such algorithms that all aim to improve some aspect(s) of how people interact with each other on social media platforms. Prominent examples are a reversion to chronological content feeds,^9^ as well as bridging‐^10^, ^11^ and intelligence‐based ranking.^12^ These proposals for alternative recommendation algorithms provide promising evidence that their implementation might lead to improvements in outcomes such as consensus‐finding and belief accuracy.^13^ While some proposals point at threats to democracy as a general justification for interventions^11^ or broadly cite the platform's importance as facilitators of public discourse,^14^ only Burton engages more deeply with how the functioning of a content recommendation algorithm could be concretely related to a healthy public sphere: his design proposal for intelligence‐based ranking rests on the argument that the formation of accurate beliefs is at the heart of a functioning civic discourse by “tapping into citizens' collective intelligence.”^12^ Providing an argument for how concrete interventions in algorithmic content recommendation systems could be derived from a normative framework is crucial to lend such interventions legitimacy,^15^ as the design of content recommendation algorithms is increasingly perceived as a tool of governance. Recent public discussions around moderation practices on large social media platforms^16^ have made it abundantly clear that ideological differences around the balance of freedom of expression versus other human and constitutional rights are directly influencing interventions in social media platform design and governance.

Against the backdrop of these societal‐level discussions, we will discuss content recommendation algorithms as a potential avenue to align social media platforms more closely with fundamental rights and democratic values, with a particular focus on the improvement of civic discourse. Here, we make three contributions that go beyond the existing arguments and proposals for alternative content recommendation algorithms in the literature: (1) We provide a comprehensive theoretical argument for the relationship between civic discourse on social media platforms and the functioning of democratic societies, and we complement Burton's approach of intelligence‐based ranking by proposing a finer‐grained picture of a healthy civic discourse that includes dimensions beyond belief accuracy, such as civility and diversity. (2) We anchor our approach in the legislative framework of the recently implemented Digital Services Act of the EU—the first of its kind that allows for the derivation of interventions in content recommendation algorithms from existing law. (3) We propose a process for how an alternative content recommendation algorithm could be designed and implemented involving stakeholders and affected communities and a situation‐dependent balance between interventions and freedom of expression, thus further enhancing the legitimacy of such an intervention.

### Civic discourse and democracy

Discourse is at the heart of any democracy as it fulfills several important functions. Classic works see public (media) discourse as central to the construction of a common identity of a collective.^17^ The discourse and its distribution via a mass media system enables complex societies to produce a feeling of belonging that is able to integrate pluralistic and complex societies. A second understanding of the public sphere follows the function of deliberation between different positions. Famously, Habermas stated that the public sphere was a necessary element in developing democracies as we know them today.^18^ By linking the civil world of the citizens and the domain of the state, the public sphere allows to openly scrutinize, debate, and potentially correct political positions.

Democratic and constitutional societies are thereby founded on the basic premise that the state and its institutions acts as a mediator for the peoples self‐governance. In this self‐governance, discourse acts as a mediator between different members of society, but also between state and citizens and is built on the assumption (which can be and mostly is counterfactual) that decisions that are being taken are based on a deliberation in which most if not all arguments and positions have been heard and weighed against each other. And this process should be inclusive in the sense that everyone can enter an ongoing debate as an equal participant. These two conditions are not always met, but in terms of democratic legitimacy, participants must be able to have them as at least probable assumptions (p. 21 f.).^19^ However, in growing and pluralistic societies, the idea that an inclusive and ubiquitous deliberation happens is not plausible. Deliberation in complex societies needs a mediator that structures the public discourse. The media system has, in this respect, an important function as it acts as such a mediator for these debates. It has, as Habermas formulates it, an informative function that bundles the vast plurality of contributions into an (more or less) informed pluralism of opinions and contextualizes them in pros and cons, in multiple perspectives and arguments. It does so by three criteria: relevance, quality, and reliability (p. 60).^19^ The classical media system of the so‐called quality media derived these criteria through a journalistic ethos; that is, by an ongoing negotiation of these values within the media system itself, but in relation to the societal function of media. This makes it hard to explicitly define these criteria formally.^20^, ^21^ Reliability creates a stable reference to presented facts, quality ensures that the media content is argumentatively understandable and presents all the relevant information and, for example, does not distort the debate by framing, and relevance ensures to link selected media contributions to ongoing political and societal debates. By doing so, the media system creates a common basis, a shared public information sphere of discussion, as it acts as a lens for a common understanding of the world — that is, the media system has also an epistemic function.^22^ Media consumers can assume that a shared core conception of reality and facts exists on which different debates are based. In social media platforms, this construction of a common information sphere has become problematic. Reliability is skewed by mis‐ and disinformation and selective debates, where facts and opinions are not separated and are not following established quality checks. Furthermore, relevance is not defined by ongoing societal and political discussions but by engagement metrics.

Because of the important function of the media system for democracies, the sector is—in most democratic states—highly regulated. By enabling the informed citizen, democratic deliberation and electoral processes should become more rational but also provide an antidote against biased perceptions or against political propaganda. This has far‐reaching consequences: for example, in Germany, the constitutional court derived from Art. 5 of the German Constitution an obligation to provide public broadcasting (see, e.g., Refs. 23, 24). While Art. 5 states that every citizen is free to inform themselves without hindrance, the constitutional court decided that public broadcasting must act as an enabler for the citizens to inform themselves. As such, public broadcasting provides the informational backbone of democratic processes by enabling informed and rational discourses to further negotiate and exchange political arguments and develop political positions. With the advent of social media platforms, this function of the media system is challenged, not only because of filter bubbles or irrational or hateful debates, but especially because it acts as a semi‐public town square on the one hand and a mediator with the function of producing a common and inclusive information space for the basis of political debate on the other hand.

## CONTENT RECOMMENDATION AND MODERATION

### Engagement maximization

**FIGURE 1:**
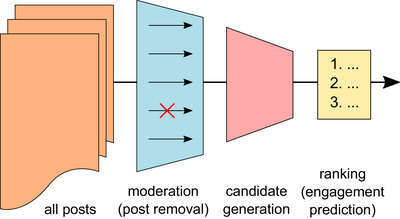
Content selection steps in a stereotypical recommender system for a social media platform (adapted from Ref. 26).

Today's social media platforms primarily serve to connect users to user‐generated content, also known as “posts.” On platforms like Twitter (now “X”), around 42 million daily active users create approximately 400 million new posts per day,^25^ while reading only a few hundred. The challenge for these platforms is to handle this information overload and select the most relevant content for each user from the available mass, while suppressing undesirable or illegal posts. This challenge is solved by recommender systems that involve several steps for content selection, including the removal of clearly illegal content like child sexual abuse material or content that infringes on copyright, the selection of a set of candidate posts, and the ranking of candidate posts into a timeline that is interspersed with advertisement (see also Figure 1).

Currently, social media platforms' business models rely on capturing user attention for advertising revenue.^27^ Their goal is to keep users entertained to maximize platform usage and ad exposure.^28^ However, the high‐level goal of user entertainment is not useful for making decisions about which piece of content to show to which user at a given point in time. To bridge this gap between high‐level outcome (user entertainment) and micro‐decisions (which piece of content to select), platforms rely on user engagement, that is, interactions such as sharing or liking,^29^, ^30^, ^31^ which is a good predictor for user retention and how much time a given user will spend on a platform.^28^ The platforms' recommender system predicts a user's expected engagement for each new post based on their past interactions with content and then selects posts that maximize the expected engagement.^28^

The strong focus on user practices as a selector for recommended content and the business model to keep user retention high, however, leads to problems for the democratic function of media systems. In Habermas' theory of deliberative democracy, he argues that separation between the private realm and the public sphere is important. Because the public sphere and its media systems have an epistemic function, higher normative demands are justified and needed at the same time. This is not true for private communication or entertainment, where a common frame of reference is not required, nor preferable. Social media undermines this separation, as it disseminates diverse content, including educational, health, news, political, commercial, art, and entertainment.^27^ For many people, social media has become their only source of information, resulting in a clash of usage contexts of the new media system. On the one hand, it serves as a means to semi‐private communication and as an infrastructure for public discourse on the other hand.

The above‐mentioned uniform optimization for engagement across all content types—which favors often highly affective, selective, and extreme content—thus imports that logic also in the realm of the public sphere. Emerging evidence shows the unintended consequences of this logic, such as the promotion of outrage,^32^, ^33^ ideological segregation,^34^ and the prioritization of content deemed “bad for the world.”^35^ Furthermore, the algorithmic mediation of information distribution according to criteria of the semi‐private approach leads to adaptation of these formats to algorithmic logic.^36^ However, changes to content recommendation algorithms to better align societal outcomes with fundamental rights and democratic values or increase the quality of recommended content may result in reduced user engagement^9^, ^14^, ^35^, ^37^ and are, therefore, not naturally aligned with platform interests.^38^ Furthermore, alternative content recommendation algorithms such as bridging‐based ranking may inadvertently censor controversial topics.^39^

### The need for system‐level interventions

There is a growing understanding that in the face of the misaligned interests of privately owned platforms and society, regulatory interventions are key to the improvement of societal outcomes.^40^ Indeed, the design of content recommendation algorithms has increasingly drawn the attention of both researchers and policymakers. Several countries have proposed legislation that mandates social media platforms to adapt their recommender systems to align more closely with democratic values and fundamental rights. The first of these new legislative frameworks to come into force was the EU's Digital Services Act (DSA), mandating large social media platforms such as X (formerly “Twitter”), Facebook, and TikTok to assess their risks to democratic values and fundamental rights, such as civic discourse and freedom of expression (DSA, Article 34^41^). Other nations are working on similar regulatory frameworks^42^ but with the enactment of the DSA in February 2024, the EU has taken the regulatory leadership. The DSA sets a comprehensive scope of relevant risks, providing a framework for interventions in social media platform design—including recommender systems—to improve societal outcomes.

The design of a social media platform includes its affordances^43^ (user action possibilities), visual design, language, terms of service, and content recommendation algorithm. This set of elements, also referred to as “choice architecture,”^44^ shapes user actions and nudges users to specific behavior.^45^ The various design components offer different ways to intervene in the platform's functioning. For example, platform companies constantly perform experiments (also called “A/B tests”) to improve their affordances and visual design to maximize user engagement and retention.^46^ As described above, this engagement maximization is also at the heart of the algorithms that select content for users.^29^, ^30^, ^31^

Until now, interventions adopted by social media platforms to prevent detrimental effects of their usage remain largely limited to the moderation of individual pieces of content that takes place before content is selected and prioritized for individual users,^47^ as illustrated in Figure 1. Analogous to offline judicial adjudication of speech rights,^47^ such interventions are driven by the need to comply with legislation that requires illegal content, such as copyright infringements or hate speech, to be removed. While content moderation is able to curb the spread of illegal content,^48^ it does not necessarily address threats to fundamental rights and democratic values that are identified as societal risks in the DSA.^41^ Moreover, the most important decisions that steer user behavior happen on the system level,^47^ where the platform's content recommendation algorithm decides which content is shown to which user.^28^ The algorithm greatly impacts how attention is distributed overall, and is, therefore, linked much more closely to societal risks. Consequently, interventions in recommender systems can be a powerful lever to address societal risks. However, the current challenge lies in translating the high‐level risks outlined by the DSA into concrete content recommendation algorithms.^27^, ^48^ Especially, as the media structure provided by social media platforms makes it necessary to not only mitigate risks but also enable the aggregative function of diverse, relevant, and reliable public opinions. This is a fundamental tension inherent to contemporary social media sites.

### Alternative content recommendation algorithms

The study of alternative content recommendation algorithms that have goals other than user engagement or retention is fairly recent. Among the few existing studies, the reduction of polarization—and, therefore, increase of the diversity of consumed content—^9^, ^13^, ^14^, ^49^ perceived harassment,^37^ well‐being,^37^ consensus and belief‐accuracy,^13^ and the trustworthiness of the consumed information^50^ are common outcomes that alternative recommender systems aim to optimize. Most prominent among the current ideas for alternative recommendation algorithms is the use of a variant of collaborative filtering to help bridge divides across diverse audiences. One implementation of such “bridging‐based ranking” (as proposed by Refs. 10, 11) suggests the promotion of content that receives positive engagement from users that come from different audience groups. Initial evidence shows that this approach is able to increase consensus,^13^ while so‐called “intelligence‐based ranking,”^12^ which entails promoting content to users that is likely to elicit belief updates that would benefit collective accuracy, is able to improve belief accuracy.^13^ On the other hand, simply reverting to chronological order^9^ or reducing the prevalence of like‐minded sources and reshares^14^, ^49^ has not succeeded in reducing polarization. However, these results have been criticized for being confounded with other, undocumented changes to content recommendation that occurred at the same time.^51^ Given the wealth of available signals that could be used for content recommendation, such as user engagement, audience diversity, and characteristics of the content itself, there is an almost infinite universe of possible content recommendation algorithms and it is not immediately clear which approach offers the most promise.

### Improving civic discourse through content recommendation

As the overall aim of this line of research is to improve societal outcomes, a rigorous foundation in politics and social change^15^ is warranted to lend legitimacy to any proposals for interventions in algorithms that likely affect hundreds of millions of people. We, therefore, suggest to anchor proposals for alternative recommender systems in existing regulatory frameworks (in particular the EU's DSA) and base them on the theoretical foundations provided by the study of the role of civic discourse for democracy. Thus, studying alternative content recommendation algorithms through the lens of improving civic discourse enables us to argue about and construct alternative content recommendation algorithms that plausibly lead to improvements in societal outcomes and are justified within the legal frameworks and processes of democratic societies.

As discussed above, civic discourse fulfills several important functions for a healthy democracy—from the construction of a common identity to the facilitation of the deliberation between different positions. Social media platforms have been shown to have a number of emergent properties or amplify individual behaviors that are detrimental or beneficial to these functions. These include the emergence of affective polarization^52^—a measure of how divided the various user groups of a social media platform are—and ideological segregation^34^—a measure of how segregated the content consumed by different user groups is. If user groups are very divided, civic discourse is endangered because conversation between different user groups is diminished, while high ideological content segregation erodes the common societal understanding of the world that is the foundation of civic discourse. Similar to ideological segregation, the spread of misinformation on social media platforms^53^ threatens to erode a common understanding of the world. Furthermore, uncivil language and hate speech that have spread on social media platforms^3^, ^54^ are a threat to civic discourse because they might cause parts of the population to leave the platform altogether, thus impoverishing the diversity of perspectives in the conversations that take place. On the flip side, social media platforms also have been reported to be positively related to civic discourse by increasing the overall diversity of news exposure,^55^ as well as knowledge^56^ of users, which can benefit civic discourse by enabling users to have more informed discussions.

Many of these outcomes have already been studied individually, and all of them are amenable to quantification, for example, by using machine learning approaches to classify the affective polarization,^57^ toxicity,^58^ hatefulness^54^ or factual density^59^ of posts, or the political ideology of users.^60^ The trustworthiness of information could be quantified by assessing the journalistic standards of news outlets, such as whether the difference between news and opinion is transparent.^61^ While Burton in his proposal for intelligence‐based ranking focuses on the formation of accurate beliefs as sole goal for an algorithmic content recommendation system, we propose that by combining the metrics that relate to different dimensions of civic discourse, a more fine‐grained measure of risk for civic discourse could be constructed—which could even include Burton's idea of amplifying good arguments. Such a measure would be composed of several components that could be given individual weights, based on societal norms about the importance of each of the composite aspects. Following the regulatory intent of the DSA, such a combined measure could then be used as alternative or complementary optimization goal for content recommendation to reduce the risk of social media platforms to civic discourse, next to or instead of engagement.

The open question, however, is how such an approach could resolve the inherent tension of social media sites as an infrastructure for the public sphere, and semi‐private communication. Diversity alone does not necessarily mean that it is inclusive of voices, presents reliable information, or signifies relevance for the audience. Classical media systems contextualized and aggregated information to form a common and commonly agreed basis for civic discourse. Even more so, the latest conflicts regarding notions of freedom of speech has shown that different political and regulatory regimes require different forms of algorithmic design and cannot solely be oriented toward a general idea of “social good.” It has become clear that content recommendation is increasingly becoming a tool of governance^62^ that has global political and ideological implications. Yet, finding implementations for recommender systems under the regulatory demands of the DSA might be a good first step toward a broader debate about the normative basis for global communication infrastructures. Such a debate, resulting in the development of concrete algorithms that translate high‐level societal norms into content recommendation decisions, is certainly an ambitious multiyear endeavor that will necessarily need to integrate the points of view of diverse stakeholders. However, below, we attempt to sketch a process that, in our opinion, would be suitable to achieving this ambition.

## TOWARD CONTENT RECOMMENDATION FOR SOCIETAL GOOD

### Multistakeholder metric construction

Quantification via metrics that measure, for example, the risk to civic discourse may create the illusion of objectivity, bearing the risk of removing political and ideological decisions from the reach of democratic processes.^63^ Therefore, as a first step in the process of designing content recommendation algorithms for societal good, it is crucial to recognize the power of metric definitions and ensure that democratic processes govern metric construction.^63^ Following democratic processes for metric construction, for example, via the involvement of stakeholders and communities affected by the design of recommendation algorithms thus enhances the legitimacy^64^, ^65^ of the resulting content recommendation decisions.

The approach of multistakeholder metric construction, originating from the field of algorithmic governance and aiming to empower individuals to design algorithmic governance mechanisms for their communities, can serve as a starting point for such community involvement.^66^, ^67^, ^68^ Lee et al.^66^ propose a process in which metrics that aim to quantify a dimension of civic discourse can be used to select pairs of posts that vary along said aspect, for example, informativeness. People are then asked to decide which of the two posts should be ranked higher in survey experiments. Such pairwise comparisons have been used to encourage moral deliberation and reach a reflective equilibrium in determining fairness principles^69^ and as a way to understand people's judgments in social and moral dilemmas in psychology and economics.^70^ Machine learning models then learn a person's utility landscape from the comparison data, based on random utility models.^66^, ^71^ As diverse people can have different utility landscapes, collective content ranking decisions by ensembles of such machine learning models provide a representative reflection of varying preferences.^66^

### Balancing risks

The implementation of alternative content recommendation algorithms to reduce societal risks is complicated by the fact that different risks may conflict with each other and an intervention to reduce one risk might increase another. This is exemplified by the trade‐off between freedom of expression and any intervention that reduces the visibility of content from a particular user. How much weight is given to specific risks to strike a just balance depends on societal values, and there is no global consensus about a just balance in this context.^72^ In addition, engagement can not be neglected as important aspect since stopping users from using social media platforms altogether is not a realistic or even desirable goal. Akin to the involvement of stakeholders in the development of new content recommendation algorithms, balancing interventions in algorithmic decision‐making with freedom of expression should involve a societal consensus‐building process to enhance legitimacy.^64^, ^65^ Recently, conjoint survey experiments have been used to resolve content moderation dilemmas in the context of misinformation^73^ and could also be employed to solicit the preferences of people regarding the balance of freedom of expression and interventions to reduce the risk to civic discourse.

Achieving a just balance between different risks is further complicated because this balance can depend on current societal circumstances. For instance, the spreading of misinformation about an ethnic minority may be evaluated differently in a region experiencing ethnic riots. Similarly, the importance of news diversity might be heightened before a major election. In addition, and analogous to states declaring a state of emergency, temporarily infringing (more) on freedom of expression might be justified to mitigate more immediate societal risks. A way to address situations where risks are weighted differently in different scenarios is scenario‐based risk assessment, commonly used in stress‐testing in the banking sector,^74^, ^75^ or when assessing the risk associated with chemical exposure,^76^ or the robustness of healthcare systems.^77^ Frameworks for scenario‐based risk assessment involve defining different stress scenarios and evaluating diverse risks in each scenario. In the context of social media platforms, different aspects of civic discourse and their trade‐off with freedom of expression can be weighted differently for various real‐world scenarios such as during elections, seeking a just balance informed by stakeholder input for each scenario. Based on this societally informed just balance, political decision‐makers could be presented with several options for the situation‐specific adaptation of the optimization goals of content recommendation algorithms. Such adaptations are then implemented and monitored following well‐defined processes that specify the scope and temporal extent of the adaptations.

## DISCUSSION AND CONCLUSION

To summarize, we propose to use the lens of healthy civic discourse to think about the design of alternative content recommendation algorithms for social media platforms. This lens provides the advantage of being rooted in the EU's recently established Digital Services Act, which explicitly mentions civic discourse as a societal good that is worth protecting in the context of algorithmic content recommendation. Functioning civic discourse relates closely to the functioning of democracy and can be related to a number of properties of discourse on social media platforms—such as content diversity, information quality, and toxicity—that can be quantified and used for content recommendation. We, therefore, hypothesize that by improving these individual aspects of discourse on social media platforms via content recommendation, civic discourse as a whole can be improved or, framed in the language of the DSA, the systemic risk that social media platforms pose for civic discourse can be reduced.

When designing alternative content recommendation algorithms and thus intervening in the status quo of engagement‐based content recommendation, trade‐offs between different risks like the risk to freedom of expression need to be considered. We hypothesize that approaches, such as multistakeholder metric construction, that build on the perspectives of diverse stakeholders such as content creators, platform companies, regulators, and users can serve to find a just balance between different risks. Next to balancing different risks, interventions in content recommendation algorithms very likely will also decrease user engagement to some extent. As that directly translates to decreased revenue from ads for social media platforms, it is not in their natural interest to implement such changes. This tension could of course be resolved by a change in the business model of social media platforms.^78^ However, we argue that a change in the design of content recommendation algorithms is a more realistic path forward in the short‐ to medium‐term future as interventions by regulatory bodies are a standard approach to align diverging economic and societal interests that have precedent in many other industries. A recent example is the change in Google's search algorithm that resulted from an antitrust case against the platform.^79^ Furthermore, a (forced) change in the platform's business model would be a much more severe infringement on the platform's freedom to conduct business than interventions in content recommendation algorithms are. For a more detailed discussion on how alternative content recommendation algorithms can serve to balance public and private interests fulfilling the criteria of necessity, effectiveness, and proportionality, we refer the reader to a recent discussion of such interventions.^80^

Furthermore, and as evidenced by recent heated discussion around content moderation practices and freedom of expression,^16^ different social norms in different parts of the world, as well as different events such as elections or a pandemic, might require different approaches to the balancing of diverse goals for content recommendation. We thus hypothesize that people's preferences regarding such a balance will vary substantially for different scenarios, thus concluding that any intervention in content recommendation algorithms needs to be able to take regional contexts into account and to be adaptive to dynamically changing circumstances. From a technical point of view, it is feasible to employ different content recommendation algorithms and moderation practices in different parts of the world and/or at different times (see, e.g., Ref. 81 for a recent example). Furthermore, it is common for globally acting corporations to be subject to different regulatory regimes in different countries. Therefore, it is not necessary to first find a global consensus on the normative basis for content recommendation on social media platforms in order to proceed with the implementation of proposals for alternative algorithms. Existing legislation such as the DSA can be leveraged for regulatory interventions without the necessity of similar legislation being enacted in the United States. Beyond the feasibility of such an approach, the “Brussels effect” could even lead to an increased speed of legislation being adopted in other countries by providing best practices and streamlined regulatory processes.

Designing alternative content recommendation algorithms that serve the societal good is a challenging process that is complicated by the increasingly difficult access to data from platform companies. Nevertheless, given the mounting evidence for a causal relationship between detrimental societal outcomes such as polarization, the erosion of trust in institutions, and the spread of misinformation and the current functioning of social media platforms, investing effort into this emerging field of research seems of high importance. With this article, we aim to provide a thorough theoretical foundation and ideas for processes that allow for the development and implementation of alternative content recommendation algorithms that aim to improve civic discourse in a legitimate way.

## AUTHOR CONTRIBUTIONS

J.L. and N.P. contributed to writing the original draft of the article.

## CONFLICT OF INTEREST STATEMENT

The authors declare no potential conflict of interests.

## PEER REVIEW

The peer review history for this article is available at https://publons.com/publon/10.1111/nyas.15359.
